# The Correction of Eye Blink Artefacts in the EEG: A Comparison of Two Prominent Methods

**DOI:** 10.1371/journal.pone.0003004

**Published:** 2008-08-20

**Authors:** Sven Hoffmann, Michael Falkenstein

**Affiliations:** Leibniz Research Centre for Working Environment and Human Factors (IfADo), Project Group “Ageing and CNS-alterations”, Dortmund, Germany; University of Cambridge, United Kingdom

## Abstract

**Background:**

The study investigated the residual impact of eyeblinks on the electroencephalogram (EEG) after application of different correction procedures, namely a regression method (eye movement correction procedure, EMCP) and a component based method (Independent Component Analysis, ICA).

**Methodology/Principle Findings:**

Real and simulated data were investigated with respect to blink-related potentials and the residual mutual information of uncorrected vertical electrooculogram (EOG) and corrected EEG, which is a measure of residual EOG contribution to the EEG. The results reveal an occipital positivity that peaks at about 250ms after the maximum blink excursion following application of either correction procedure. This positivity was not observable in the simulated data. Mutual information of vertical EOG and EEG depended on the applied regression procedure. In addition, different correction results were obtained for real and simulated data. ICA yielded almost perfect correction in all conditions. However, under certain conditions EMCP yielded comparable results to the ICA approach.

**Conclusion:**

In conclusion, for EMCP the quality of correction depended on the EMCP variant used and the structure of the data, whereas ICA always yielded almost perfect correction. However, its disadvantage is the much more complex data processing, and that it requires a suitable amount of data.

## Introduction

Psychophysiological research requires the acquisition of small signals. Hence a big problem in this research area is the sensitivity of these signals for artefacts. Despite the reduction of technical artefacts in the recent years, the impact of biological artefacts still represents a considerable problem. Especially in the acquisition of the electroencephalogram (EEG) they play an important role, since the recorded signal is, compared to other biosignals, very low. One of greatest nuisances are those artefacts resulting from oculomotor activity. These artefacts are almost inevitable because subjects cannot well control spontaneous eye movements or blinks. Further, the instruction to inhibit eye movements or blinks may seriously distort brain activity [1]. Several methods have been developed to cope with the problem of ocular artefacts. The most popular approach is the correction of ocular artefacts by means of regression analysis.

In general, in regression based approaches propagation factors are calculated to estimate the relation between one or several EOG channels and each recorded EEG-channel [2]. These propagation factors are usually estimated by least squares regression. The eye movement correction procedure [EMCP, 2] is an example for such an approach. The rationale of the procedure is:

1. Raw averaging: averaging of all trials with respect to an event for each EEG and EOG-channel to estimate the event related variation for the EEG and EOG-channels
2. Raw average subtraction: subtraction of the raw averages from every single trial to estimate the activity at an electrode site, for each trial, that is not event related.
3. The propagation factors are computed by linear least-square regression, whereby the EOG-data serves as the independent variable
4. Correction: the derived propagation factors are used to correct the raw EEG data by subtraction of the EOG-values scaled by the propagation factors

A theoretically different approach for the correction of ocular artefacts is based on the assumption of a component model. The goal of these procedures is to decompose EOG and EEG into spatial and temporal distinguishable components. After identification of components constituting ocular artefacts, the EEG is reconstructed without those components. The most popular example for a component based procedure is principal component analysis (PCA) [e.g. 3]. Another technique is the correction by using a dipole model [4]. With this multiple source eye correction (MSEC) method ocular artefacts are modelled by moving dipoles of the eyes, and this activity is subtracted from the EEG.

A more recent method is Independent Component Analysis (ICA), which is an approach for the solution of the blind source separation (BSS) problem [5]. It is not only a correction procedure, but a more general approach for multivariate data analysis. The general model of ICA is that the observed signals *x* are constituted by linearly mixed (*A*) sources *s* (*x = As*). These are unknown and mutually statistically independent. Since mixture and sources are unknown, the inverse of the mixing matrix *W* has to be estimated blindly. This leads to a *solution u = Wx*, where *u* are the estimated sources. The estimation of W is based on minimizing a cost function that enforces statistical independence.

ICA is, like PCA, a method for decorrelating data, but whereas PCA uses only second order statistics and assumes the underlying sources to be orthogonal, ICA uses higher order statistics. Concerning the EEG, it is assumed that the recorded signal is a linear mixture of unknown sources within the brain. Because the sources and therefore the mixture are unknown, they need to be estimated. The basic assumption of ICA, that the sources are statistically independent while the mixture is not, is neuroanatomically and neurophysiologically plausible, since cortical (and other) areas are spatially distinct and generate a specific activation, but correlate in their flow of information [6]. Several algorithms have been developed to solve the BSS problem. In the present study the extended infomax [7] (infomax = information maximization) algorithm was used. Infomax had already been shown to be a reliable method for the decomposition of multi-channel EEG data [8]–[12]. The basic correction procedure with ICA is shortly described as follows (without pre-processing steps):

1. Conduction of ICA by an appropriate algorithm
2. Identification of blink-like components (e.g .by time-course of activity, scalp topography)
3. Removal of the blink-component and backprojection of the remaining components by x_clean_ = W^−1^u [11]

Although several studies have compared different algorithms with respect to their performance in artefact correction [13]–[16], only few attempts have been made to compare the different correction procedures concerning their impact on the event-related-potential directly.

The present study investigated to what extent the EEG was still contaminated by eyeblink related activity after application of different artefact correction procedures. Since it is an open discussion which procedure is appropriate in general for the correction of ocular artefacts, two widely used approaches with different theoretical background were chosen to investigate former question. The regression based approach (EMCP, [2]), is the classical well established algorithm. It was conducted with (EMCPs, which is the original algorithm, [2]) and without raw average subtraction (EMCP w/s), since omitting the subtraction is the classical regression approach. It was likely to yield different results as the EMCPs. The component based approach was the extended infomax algorithm for Independent Component Analysis [7], [17], [18].

To investigate the remaining artefact activity in the EEG subsequent correction, the EEG-data was time-locked to blinks, because this approach highlights even very small residual artefacts [19]. Also in real experimental situations some subjects tend to blink time locked to events; hence the approach appears to be realistic.

The present study only dealt with spontaneous eyeblinks, since with the approach of blink time locked data it is possible to derive a good estimate of only blink-related activity. Hence the derived blink-related potential is not contaminated by processes resulting from some kind of experimental paradigm (i.e. stimuli or responses).

One problem is the choice of an adequate measure to estimate the residual impact of the blink artefact to the EEG. Here linear regression, because of its simplicity in calculation appears to be the first choice. However, linear regression requires linear dependent and normally distributed data. This may not always be the case with EEG and EOG data. As already mentioned blink-time locked averages for each correction procedure were calculated. Unlike Berg [19] not only ERPs (i.e .the residual activity at blink-time) were compared across methods, but also the mutual information of blink-locked data of the vertical EOG and relevant EEG-channels. This approach was chosen to quantify if the corrected EEG still contains information due to eyeblink activity. Compared to second order statistics (like covariance and hence correlation) mutual information is a more sensitive measure for the statistical independence oft two random variables. It is a more general measure for estimating not only linear dependencies, but also dependencies of higher order [20]. Further it is independent of the distribution of both tested variables. In contrast, correlation requires the variables to be gaussian if their independence is to be tested.

However, for EEG data it has not yet been systematically tested whether the distributions of the values in the single channels are always gaussian. In case of non-gaussian distributions correlation, and validation by linear regression are not appropriate measures of independence, i.e. the contribution of the eyeblink to the corrected EEG.

It is assumed that after correcting the EEG it should share less information with the EOG than the raw uncorrected EEG data, since the removed blink signal carries most of the mutual information in blink-time locked data. At first glance this seems circular, since ICA reduces mutual information, but ICA and correction by removal of one component must not be confused. ICA minimizes the mutual information between the estimated sources. The correction is done by the former described backprojection x_ clean_ = W^−1^ u, which is a linear transformation like with the regression approach. The amount of reduction of mutual information depends on how much information the removed component contributed. If it was high, the data would be less correlated, and hence the mutual information between EOG and EEG reduced. But this is also true for the regression approach: The propagation factor depends on the correlation between EEG and EOG. A high propagation factor means that EEG and EOG share much variance. Hence by subtraction of the weighted proportion of the EOG from the EEG, they would be also de-correlated.

Since the final goal of a correction procedure is not to make EEG and EOG independent, but rather to eliminate eye blink activity from the EEG, it is necessary to additionally have a look at the time courses of activations in the EEG following correction. With respect to this, less blink-related EEG activity, i.e. transient(s) showing a similar time-course as the eye blink data, should be visible at the selected electrode positions. In summary, mutual information and blink-related activity was used to assess residual blink-related activity after application of different correction procedures on real data. The applied procedures were EMCP with raw average subtraction (EMCPs), EMCP without raw average subtraction (EMCP w/s) and an algorithm for Independent Component Analysis (extended infomax).

Additionally, simulated data were contaminated with eye blink artefacts and subsequently corrected to assess the residual activity remaining following correction. This simulation was added since with empirical data, the “true” sources are not known, and hence the goodness of correction can only be estimated by an indirect measure. In the case of the present study this was done by estimating mutual information. However, this provides only an indirect approach and the ground truth is unknown. This is not true for a data simulation. Here the clean, uncontaminated data are known and it is possible to estimate precisely the correction error of the procedures. Moreover, a simulation provides the advantage that it can be tested whether hypothetical occurring residual activity after correction is due to over-correction or not. Since it is not desirable to benefit one correction procedure by the type of model used for simulation, two models were used for the present study: One model that generated eyeblinks by assuming a component model, and a model that generated eyeblinks by means of regression (i.e. propagation).

## Results

### Real data

**Figure 1:**
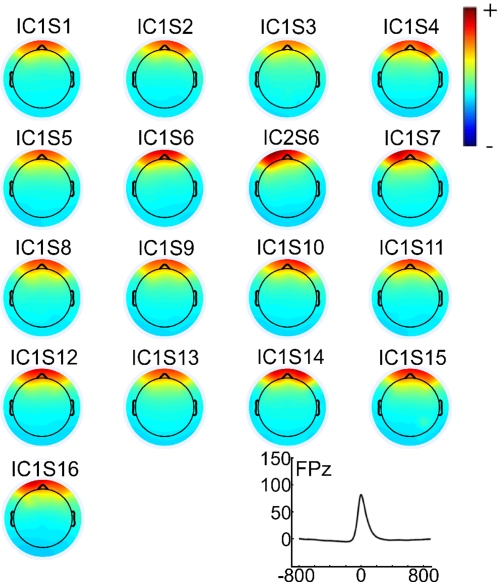
Topographic maps of the projection of blink components. Upper left: Grand average of activation of the blink-components back-projected to FPz. Right: Scalp topographies of the blink components (ic) for each subject (s). Note that ICA revealed for one subject (s6) two blink related components. The colour-map legend does not contain values since the ICA topographies represent arbitrary values.

The mean blink rate of the participants was 4.19/min (s = 3.89). The average blink amplitude was 215.28 µV (s = 40.29) at SO2. The blink amplitude and its variance correlated significantly with the mean mutual information prior to correction (r = .71; p<.01 and r = .65; p<.01 respectively). Independent Component Analysis revealed, for every participant, blink related components showing the typical time-course and projection to frontal electrode positions of blink related activity that is observable in the uncorrected EEG (Figure 1). For every subject a full dimensional ICA decomposition was conducted (i.e. without PCA preprocessing). The k-means procedure as implemented in EEGLAB clustered the components clearly into blink components and other activity. On average the blink components accounted for 99.58% variance in the time window from −50 to 50 ms around the maximum blink excursion. For one subject ICA yielded two blink related components (Figure 1).

#### Mean mutual information

With respect to the mutual information the overall repeated measures ANOVA with the factors correction procedure (extended infomax, EMCPs, EMCP w/s, raw data) and electrode (FPz, FCz, C3, C4, PO7, PO8, Pz, Oz) revealed a main effect of procedure (F(3,45) = 191.95; p<.001; *ε* = .72), a main effect of electrode (F(7,105) = 3.91; p = .006; *ε* = 0.61) and an interaction of procedure and electrode (F(21,315) = 5.58; p<.001; *ε* = .249).

The contrast of extended infomax vs. EMCPs revealed a significant main effect (F(1,15) = 13.248; p = .002), showing that mutual information was higher for EMCPs than for infomax. There was no significant effect of electrode position (F(7,105) = 1.269; p = .294; *ε* = .51) nor a significant interaction of procedure and electrode position( F(7,105) = 2.39; p = .07; *ε* = .52).

**Figure 2:**
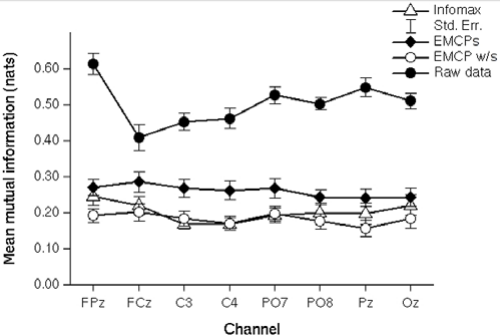
Real data: Mean mutual information of corrected EEG-channels and uncorrected vertical EOG. Mutual information (1 *nat*≈.44·1/(log 2) bits) is highest for uncorrected data, lower for EMCPs, and lowest for Infomax and EMCP w/s corrected data. Infomax = extended infomax.

The contrast extended infomax vs. EMCP w/s revealed a significant main effect of procedure (F(1,15) = 11.75; p = .004) showing that the mean mutual information was higher for infomax than for EMCP w/s, and a significant interaction of procedure and electrode position (F(7,105) = 4.01; p = .005; *ε* = .6), showing that the effect of procedure varied with the electrode position, being higher at frontal positions and lower at occipital positions (Figure 2). The effect of electrode position showed a tendency to significance (F(7,105) = 2.59; p = .06; *ε* = .491).

Further the mean mutual information was significantly lower after EMCP w/s than after EMCPs (F(1,15) = 26.59; p<.001). There was no significant effect of electrode position (F(7,105) = 1.13; p = .35; *ε* = .74) or interaction of procedure and electrode position (F(7,105) = .32; p = .82; *ε* = .44).

In summary the mean mutual information of vertical (uncorrected) EOG and corrected EEG-channels was significant lower following application of extended infomax and EMCP w/s than after EMCPs (Figure 2). Further it differed significantly between extended infomax and EMCP w/s. Mutual information varied with electrode position, being larger at frontal and occipital positions, and smaller at more central and lateral positions (Figure 2).

#### Residual activity

**Figure 3:**
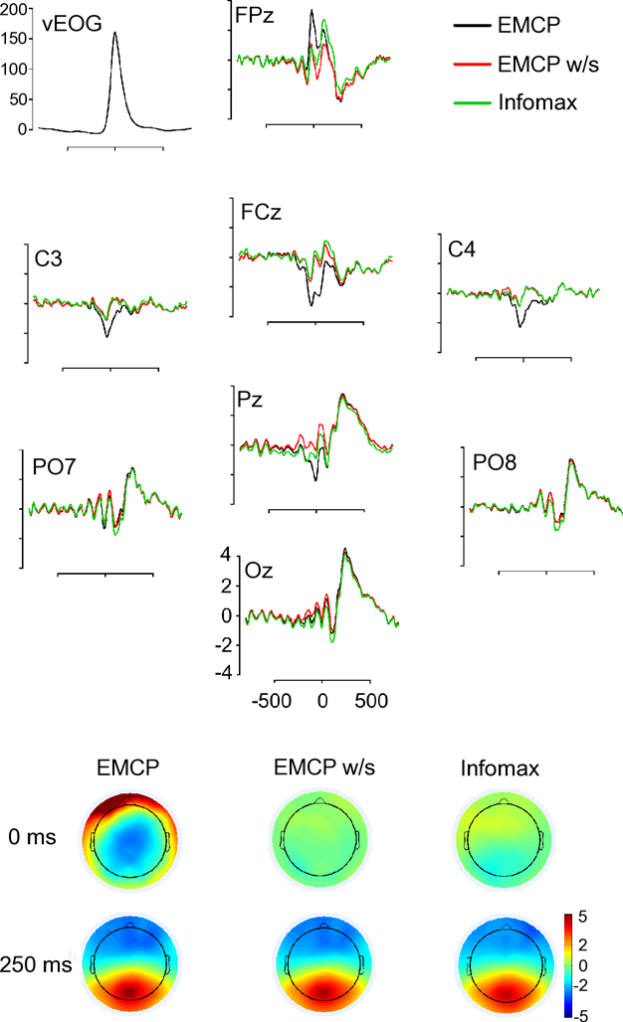
Blink-time locked grand averages of corrected data. Upper figure: Grand averages of activation (µV) of data corrected by Infomax, EMCPs and EMCP w/s. Lower figure: Topographic maps (spherical spline interpolation) of the activation subsequent correction at the time point of maximum blink excursion (0 ms) and 250 ms after it. Infomax = extended infomax. vEOG = (|SO2|-|IO2|)/2

A blink-related positivity was observable (Figure 3), that peaked at about 250 ms after the blink maximum. This positivity was observable after application of either correction procedure (infomax: t(15) = 8.62; p<.001; EMCP w/s: t(15) = 7.08; p<.001; EMCPs: t(15) = 7.20; p<.001). It did not differ significantly between the different correction procedures (F(2,30) = 1.34; p = .27; *ε* = .57).

Figure 3 and Animation S1 (suppl. material) show the blink-related potentials and the corresponding topographic maps. The topographic maps support the results of the mean mutual information analysis, revealing residual positive activation at the former blink-maximum after either correction procedure.

### Simulated data

**Figure 4:**
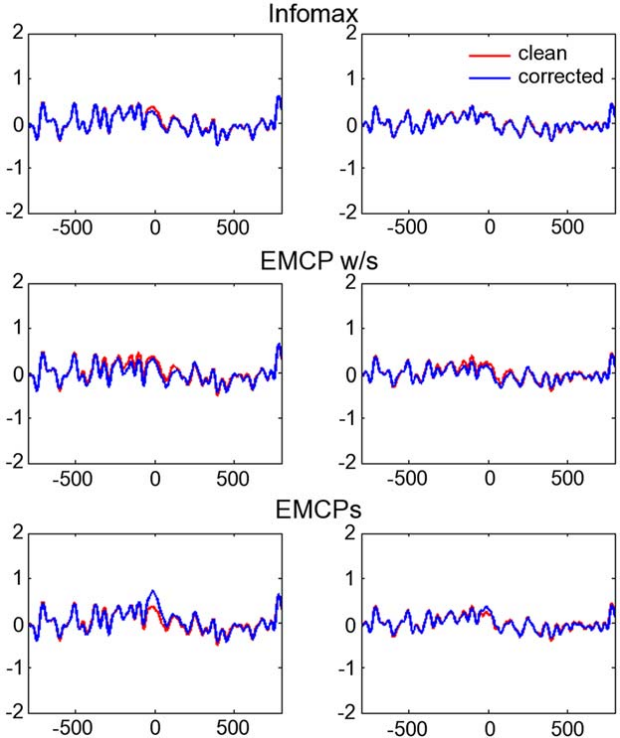
Component model. Averages time-locked to the maximum blink excursion (x-axis: time (ms); y-axis: µV). Only two channels are plotted (left and right column respectively). Red lines: clean, uncontaminated data; Blue lines: corrected data. Note the difference in the scaling for the EMCPs-corrected data. Also note that the real names (like “FCz”) are not informative, since they don't have spatial information. The reason is that the components were selected randomly for the mixing, as well as the values for the mixing matrix.

The results indicate a differential impact of the tested procedures on the simulated and real data. With respect to the eyeblinks simulated by the **component model**, the results are almost in line with those from the real data. The residual activity at the former maximum blink excursion was largest for the data corrected by EMCPs, while ICA-corrected data as well as data corrected by EMCP w/s resemble almost perfectly the time course of the clean uncontaminated data. Figure 4 shows the blink-time locked averages for the clean (i.e. uncontaminated) data and data corrected by extended infomax, EMCPs and EMCP w/s.

**Figure 5:**
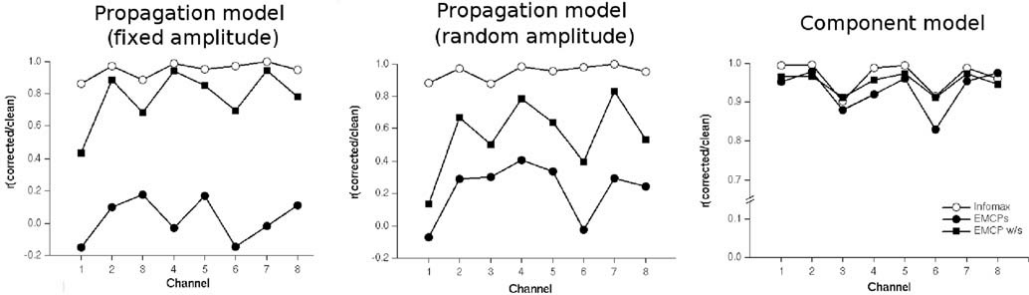
Correlation of clean uncontaminated data and corrected data. The figure shows the correlation between clean, uncontaminated data channels and corrected data channels for each simulation type.

This is supported by the correlations between the clean, uncontaminated data and corrected data (Figure 5).

As regards the data contaminated by the **propagation model**, the results show that the residual activity at the blink time maximum was larger for both EMCP procedures than for ICA-corrected data. ICA corrected data resemble almost perfectly the time course of the clean uncontaminated data.

**Figure 6:**
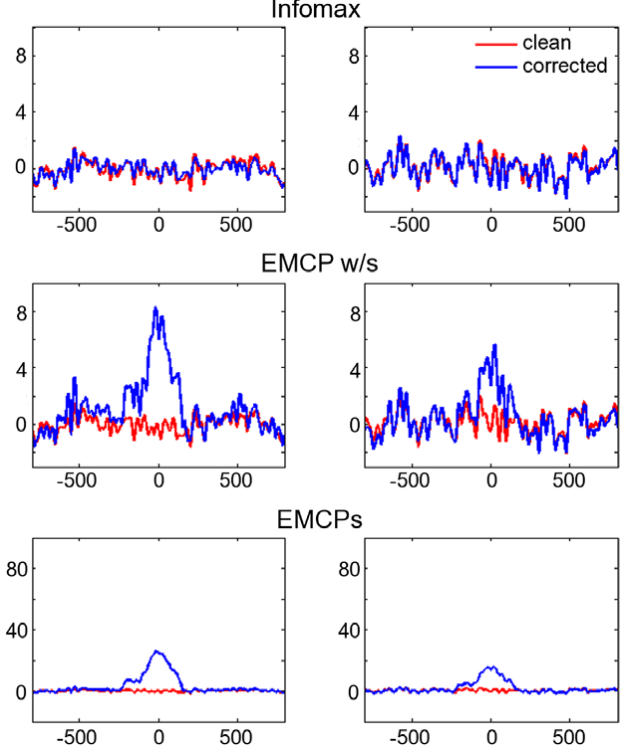
Propagation model with randomly varying blink amplitude. Averages time-locked to the maximum blink excursion (x-axis: time (ms); y-axis: µV). Only two channels are plotted (left and right column respectively). Red lines: clean, uncontaminated data; Blue lines: corrected data. Note the difference in the scaling for the EMCPs-corrected data. Also note that the channels for the propagation model have been chosen randomly, as well as the propagation factors used for simulation of blinks. Hence the real names (like “FCz”) are not informative, since they don't have spatial information anymore.

**Figure 7:**
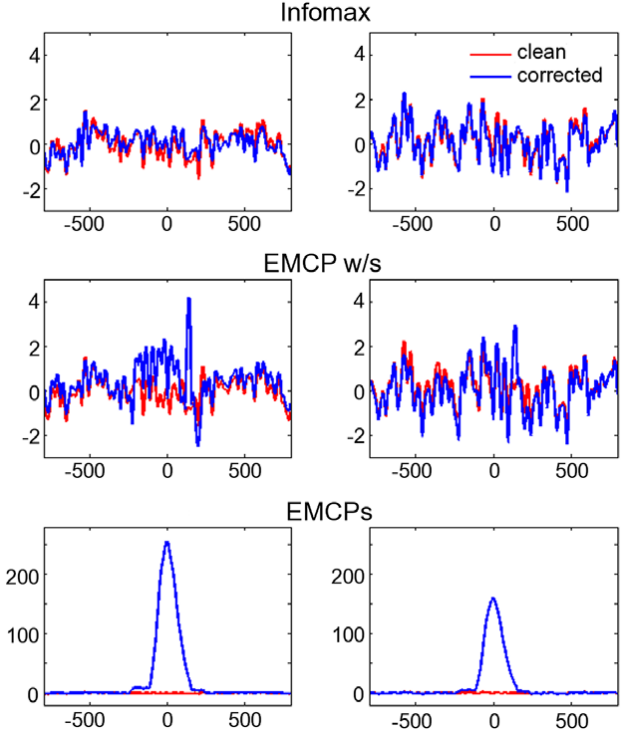
Propagation model with constant blink amplitude. Averages time-locked to the maximum blink excursion (x-axis: time (ms); y-axis: µV). Only two channels are plotted (left and right column respectively). Red lines: clean, uncontaminated data; Blue lines: corrected data. Note the difference in the scaling for the EMCPs-corrected data. Also note that the channels for the propagation model have been chosen randomly, as well as the propagation factors used for simulation of blinks. Hence the real names (like “FCz”) are not informative, since they don't have spatial information anymore.

This pattern was present in simulated blinks with randomly varying amplitude (Figure 6) and in blinks with constant amplitude (Figure 7). The former revealed remarkable residual activity for the data corrected with EMCP w/s.

With respect to the correlation between corrected and clean data this data pattern was also present: It was largest ICA corrected data, smaller for EMCP w/s and even smaller for EMCPs (Figure 5).

## Discussion

The present study shows that after application of methods for the correction of ocular artefacts there still remains activity at the time point of the eyeblink artefact in real data. This activity shows an occipital topography that peaks at about 250 ms after the blink maximum excursion. This result replicates the findings of Berg [19]. The residual cannot simply be explained by some kind of overcorrection by the correction procedure, since it remains after application of either procedure, and it is not visible in the simulated data. Further mutual information of vertical EOG and EEG as well as the residual activity at the maximum blink excursion shows differential effects with respect to the applied correction procedure and electrode site. These effects were also present in the simulated data.

### Real data

With respect to the mean mutual information extended infomax and the regression approach *without* raw average subtraction (EMCP w/s) yielded almost the same results. However, though there was a significant difference between extended infomax and EMCP w/s. But though this difference was statistically significant, the difference was, in terms of absolute values (Figure 2 & 3, suppl. Animation 1), quite marginal. It could be interpreted as a result of the different number of eyeblinks of the subjects. Since ICA is a statistical procedure, it requires a source to show frequent activation, if should be reliably extracted. An alternate interpretation could be that EMCP w/s lead to an overcorrection of frontal and occipital channels. Another possibility is that extended infomax only removed the pure blink activity, while there remained activity accounting for muscle or vertical eye movements accompanying the reopening of the eye. This would increase the difficulty to identify components accounting for ocular artefacts of this type, since only blinks are unambiguously identifiable. For the regression approach *with* raw average subtraction (EMCPs) the residual mean mutual information was higher, and it was highest for uncorrected data. After application of EMCPs the blink-related averages and topographic maps of the activity at the former blink maximum showed large residual activity. Interestingly, for one subject ICA yielded two components accounting for blink activity (Figure 1, s6). This indicates that under certain circumstances ICA might result in a higher dimensional decomposition for a component in the EEG as predicted. This has to be object of further investigations. Following either correction procedure a large positivity in the time-window at about 250 ms after blink maximum was observable. This replicates the findings of Berg [19]. He suggested that this residual has physiological origins and results from the re-opening of the eye, that induces a strong change of the visual input and hence a visual evoked ERP. As a conclusion, this residual should be taken into account if the EEG is corrected by any kind of correction algorithm. In further studies it should be investigated to what extent this residual contributes to the estimated ERP, and whether it can be removed by ICA as well.

### Simulated data

Correlation and residual activity varied differentially between the different correction procedures. For ICA the procedure yielded the same results for both models (i.e. propagation model, component model): It showed almost the same residual activity like the uncontaminated clean simulation data. Also the corrected data correlated highly with the clean uncontaminated data. This was also true for EMCP w/s. However, this was not the case for EMCPs. Correlation (Figure 5) and residual activity (Figures 4, 6, 7) were always higher than for ICA and EMCP w/s and EMCPs.

In the case of a constant blink amplitude EMCPs seemed to fail, since the blink-related average still showed the same time-course of activity like the uncorrected data. The correction was better for the component model, and also if the blink amplitude varied. This was the case either for the component model and one propagation model. EMCP w/s showed comparable results to ICA, if a component model was assumed and if the blink amplitude did not vary strongly in the propagation model. However, with varying simulated blink amplitude even EMCP w/s was slightly inferior to ICA.

Finally, following correction by either correction procedure there was no positivity following the blink maximum. This indicates indeed that this potential is a physiological potential resulting from the re-opening of the eye.

The results may be due to the fact that if the blinks do not vary in their amplitude and duration, the vertical EOG at blink time point represents a good estimate of the “true” blink signal. If they vary, the estimation by linear regression can be a rather imprecise approximation. This may be an explanation why EMCP w/s and ICA lead to analogous results in the real data: Spontaneous blinks usually do not vary strongly in amplitude and duration, As a consequence the regression error is small. Hence, the question arises which simulation represents an appropriate model. With respect to the data of the present study it seems as if the component model fitted better, since the results of the correction procedures was quite analogous for simulated and real data.

In summary the data simulations support the findings in the real data. The impact of the ICA procedure was equivalent in the propagation model as well as in the component model. In both cases the ICA-corrected data resembled almost perfectly the time course of the clean uncontaminated data. This was not the case for EMCP-corrected data. While a large residual activity appeared in both models (i.e. blinks realized by a component model and those constructed by a propagation model) the simulations reveal also, that the residual varies with the variation of the blink amplitude.

Interestingly the results for the mean mutual information correspond with the results of the simulation. This supports the idea that mutual information can be used as an evaluation criterion. Its advantage is the independence of the distribution parameters of the electrical activity of EEG and EOG.

It may be argued that the paradigm of blink-time-locked data is artificial. However, in EEG experiments participants often blink in a regular pace shortly after a response or a stimulus. Further the blink rate varies with different cognitive or activation states and between participants. Based on the data of the present study, it could be stated that the conduction of EMCPs is always obsolete, but this conclusion cannot be generalized. The present study did not test the impact of either correction procedure on other event-related potentials. Here, one question is, what happens if EMCP w/s is applied to data in which eyeblinks occur temporally close to event-related potentials. This is why Gratton et al. [2] developed the procedure with raw average subtraction: it should avoid the distortion of the ERP by subtraction of the ERP prior calculation of the propagation factors. In conclusion it seems, as if there were optimal conditions for both options for calculating the EMCP. However, it is desirable that a correction procedure should produce adequate results irrespective of blink rate, time-course, or time-point of blinks relative to the experimental event or reaction. Hence further investigation is necessary (e.g. by variation of blink frequency, blink-time point) to disentangle the “optimal” conditions for each of the two variants.

The researcher has hence to decide which regression approach is the adequate solution for his data. The data structure, e.g. the relation of blinks to events, may be different across subjects and conditions, hence different regression procedures may be adequate for different subjects and conditions. This raises the problem of the appropriateness of conducting two different correction procedures within one analysis framework. This can only be approved if both procedures lead to mathematical equivalent results.

In contrast to the regression approach used in the present study, ICA has the advantage that only few assumptions about the underlying structure of the data have to be made [21]. Regardless of the blink structure, ICA seemed to yield almost perfect correction, which is a strong argument for conducting ICA.

However, there are also some drawbacks concerning the computation of ICA. On the technical side, compared to regression procedures, the computational load is very high. Depending on hardware, data length, and number of channels, the computational time is certainly much longer than for regression analysis. Also there are some mathematical constraints to the application of ICA. The sources are assumed to be statistically independent. This means, in terms of EEG, that it is assumed that spatially static sources generate temporally dissociable time-courses of activation. However, neural networks are often overlapping, but they might generate different patterns of activations. Thus, with EEG data temporal ICA is mostly conducted. Further, ICA requires at least as many simultaneously recorded signal mixtures (e.g. sensors) as there are signal sources (e.g. voices, neural networks). Third, there must not be more than one gaussian source. And finally, the influence of random noise has to be kept as low as possible, since the basic ICA model, which is also assumed by infomax, assumes no noise. For data with additional noise other algorithms have been developed [22]. Also a minimum of data points is required to estimate a stable (i.e. reliable) decomposition. Since ICA is a statistical procedure it is sensitive to random noise. If there are only few artefacts of one kind it is possible that the decomposition fails. This is what is indicated by the significant difference between extended infomax and EMCP w/s. Hence it follows, what sounds at first glance counterintuitive, that the decomposition is the better, the higher the number of blink artefacts.

Finally there are many possible solutions (i.e. algorithms) for the BSS problem; the choice of an algorithm depends on the data and the assumptions about its underlying factors. Up to now it has not been tested systematically which algorithm may be the most appropriate one for EEG data.

Another drawback is that the identification of blink-components is usually done by visual inspection, i.e. it may suffer from a subjective bias. However, in the present study the blink components were not only identified by visual inspection, but by cluster analysis and the percent of variance the components accounted for in a defined time-window around the maximum blink excursion. The blink-components, that showed blink-like topographies and time-courses, accounted for about 99% of variance. Further the k-means procedure was able to combine these components into one cluster.

In conclusion the results show that extended infomax and EMCP w/s may lead to an almost analogous impact on mutual information of vertical EOG and EEG channels as well as the residual activity that remains at blink time maximum. However, while the optimum use of EMCP appears to depend on the variance of blink activity, ICA is independent of a certain data structure and, what is more important, it is not restricted to the removal of blink artefacts. It provides a general framework for artefact removal and analysis of EEG-data. Furthermore, what is most important, the performance of ICA seems to be independent of the model that is assumed about the generation or propagation of the eyeblink signal. Another important result is the late blink related positivity that was observable after either correction procedure.

However, the present results cannot be extended as a general conclusion against regression based approaches. It has to be stated that a limitation of the study is the restriction to eyeblinks. Further investigations are necessary with respect to vertical and horizontal eye movements. There are several regression methods that have been shown to lead good results [23]–[26]. However, aim of the present study was not to evaluate regression procedures in general, but rather to evaluate the impact of the EOG on the EEG after correction with two common used procedures. Nevertheless, further research is necessary to evaluate systematically the performance of different correction procedures (i.e. component models, regression approaches), since the discussion about that topic has not been settled.

Another constraint, however, is the residual activity after the blink maximum: If only components accounting for blink activity are subtracted, there might remain activity in the EEG that is blink related and therefore can be defined as artefactual. Hence, the correction of ocular artefacts with ICA should not focus solely on components accounting for eyeblinks or horizontal eye movements. ICA, because of its power in the decomposition of multivariate data, may be a suitable tool to remove the mentioned positivity as well. This has to be topic of further investigations, since it is an important issue: If conducting ICA it might be (e.g. due to data quality or violating the basic assumptions of ICA) that during conduction of ICA activity that is temporal correlated with the blink component is removed as well since the decomposition might not have been perfect. However, the present results indicate indeed that ICA decomposes a pure “blink signal” which is independent of the residual, since the residual does not occur in the corrected simulation data.

Here further investigation is necessary to reveal the components corresponding with ocular artefacts. Also more detailed investigations of the dynamics of different kinds of ocular activity are necessary. Spontaneous, voluntary and reflectory eyeblinks differ in their neural sources and the factors inducing them [27] , for example activation, cognitive load, intention, physical irritation or pathological reasons (e.g. blepharospasm). ICA may be a powerful tool to disentangle the different sources in those different blink conditions. It cannot simply be regarded as a correction procedure. Because the components accounting for artefacts and those accounting for neural activity are independent of each other, a correction of the EEG would be obsolete, if the focus was on the functional significance of the derived independent components.

Finally, our conclusion is that ICA is a powerful tool for the correction of blink artefacts. However, there exist several possible algorithms for conduction of ICA. The performance of these algorithms has yet systematically to be tested. Also previous studies have shown that besides EMCP, there exist a couple of powerful correction procedures, as well as regression based, and component based approaches. Here a systematic re-evaluation is necessary to evaluate the gain of new (i.e. ICA) correction procedures.

## Materials and Methods

### Subjects

Seventeen subjects (10 females) aged from 19 to 30 years (m = 22.7, s = 3.6) participated in the study. Participants were healthy undergraduate students who received course credits for their participation in any psychological experiment, which is part of the curriculum in the German academic studies of psychology. No grading was assigned. Participation was absolutely voluntary. All participants were right-handed and gave written informed consent before participation. The study was approved by the ethics committee of the Institute for Occupational Physiology at the University of Dortmund. Participants had normal or corrected to normal vision. The data of one participant had to be rejected because of too many artefacts.

### Stimuli and procedure

Participants were seated in a light and sound dimmed room in front of a standard CRT monitor. The distance of participant and monitor was about one meter. Their task was simply to focus a white fixation cross presented in the centre of the monitor. This was necessary in order to minimize eye-movements; furthermore this is a common procedure in experiments in which the EEG is acquired during a reaction-time task. The participants were instructed not to move if possible; they were not instructed to avoid eyeblinks. After ten minutes the EEG acquisition was stopped.

### EEG-recording

EEG was recorded from 57 channels relative to average reference using a QuickAmp 72 (Brain Products). Channels (FPz, FP1, FP2, AFz, AF7, AF3, AF4, AF8, Fz, F7, F3, F4, F8, FCz, FT9, FC5, FC3, FC1, FC2, FC4, FC6, FT1, T7, C5, C3, C1, C2, C4, C6, T8, CPz, CP5, CP3, CP1, CP2, CP4, CP6, Pz, P7, P3, P1, P2, P4, P8, Oz, PO9, PO7, PO3, PO4, PO8, PO1, Oz, O1, O2, I1, I2, Cz) were positioned following the 10-20-system (Jasper, 1958). Additional electrodes were positioned at the mastoids (M1, M2) and four electrodes were used to record the electrooculogram (EOG) from positions below (IO2) and above the right eye (SO2) and from the outer canthi (LO1, LO2). All channels (EEG,EOG) were recorded with respect to the same reference (average reference). Impedances were kept below 10 kOhm. Sampling rate was 500 Hz (no highpass, lowpass 135 Hz).

### Analysis

#### Preprocessing

Data analysis was conducted offline using the Brain Vision Analyser software (v1.05, Brain Products) for pre-processing and implementation of the eye movement correction procedure [EMCP, 2]. Matlab (The Mathworks) and EEGLAB v5.03 [28] were used for further processing and ICA. After importing the data into the Vision Analyzer software, raw data were band-pass filtered (0.5–30 Hz) using a phase-shift free butterworth filter (12 dB/Octave). Subsequently a threshold algorithm was conducted to detect eyeblinks, and the data were segmented time-locked to the maximum blink excursion (−800: 1000 ms). Following this a baseline correction was made (−800:−500 ms). Finally, the continuous raw data were cleaned from occasional non blink-related artefacts by visual inspection.

#### Ocular Correction

*Regression analysis*

For the calculation of the EMCP vertical and horizontal EOG (SO2, IO2, LO1, LO2) were used and the regression was calculated with (EMCPs) and without raw average subtraction (EMCP w/s). For the simulated data only the simulated vertical EOG channel was used since only blinks were simulated.

*Independent Component Analysis*

For the ICA procedure the filtered continuous raw data and blink-related *uncorrected* segments were exported to EEGLAB.

ICA was conducted using extended infomax [7] as implemented in EEGLAB (default parameters, except: maximum number of iterations = 800) and the derived weight matrices (i.e. unmixing matrices, *W*) were applied to the blink-time locked data. All data (EEG and EOG) were included in ICA. Subsequently the derived independent components were clustered (k-means) using their topography and blink-time-locked averages of activity. Two main clusters (i.e. components accounting for blink-and EEG-activity) were assumed and defined for clustering. Components representing blink artefacts were identified by visual inspection of component activations, projections of the components to the scalp (inverse weight matrix for the component) and by the variance accounted for [8], [13] in the time window from −50 to 50 ms around the maximum blink excursion. It was assumed that independent components accounting for blink artefacts show the typical blink-like time-course and topography and account for the most variance in the EEG data in the time window from −50 to 50 ms around the maximum blink excursion. Finally, the components representing blink artefacts were removed and the remaining independent components projected back.

#### Artefact processing

Prior removal of blink components a semi-automated artefact rejection procedure was applied as implemented in EEGLAB to remove residual artefacts not detected by visual inspection. These procedures are highly efficient in detecting linear trends and improbable data segments [29]. From the EMCP-corrected data the same segments were removed as from the ICA-corrected data. Hence the segments used for further averaging and statistical analysis were the same for both procedures.

#### Dependent variables

*Mutual information*

For each subject mean mutual information was calculated between the uncorrected vertical EOG and each predefined *corrected* EEG-channel (FPz, FCz, C3, C4, PO7, PO8, Pz, Oz). These channels were chosen because they are the most frequently used channels for EEG research, and because they cover the nearest and most remote scalp positions relative to the eyes. Also the mean mutual information of the uncorrected vertical EOG and the *uncorrected* EEG-channels was calculated.

*Residual activity*

The residual transient activity in the time window from 200–300 ms after the blink maximum was detected by a peak detection algorithm.

### Statistics

Initially an overall factorial analysis of variance with repeated measures was calculated for the mean mutual information. Factors were electrode position (Fpz, Cz, C3, C4, PO7, PO8, Pz, Oz) and correction procedure (EMCPs, EMCP w/s, extended infomax, raw data).

One dependent variable was the mean mutual information for each uncorrected vertical EOG and corrected EEG-channel pair. For the raw data the uncorrected EEG and uncorrected vertical EOG pairs were used. Two factorial analyses with repeated measures were calculated to compare both regression variants with extended infomax.

Finally paired t-tests were conducted with respect to residual EEG activity that hypothetically occurs in the time window from 200–300 ms after the blink maximum (according to Berg, 1988). Greenhouse-Geisser adjustment to the degrees of freedom was performed for effects with df >1. In that case Greenhouse-Geisser epsilon, uncorrected F-values, and corrected p-values are reported.

### Simulation

For simulation of eyeblinks in EEG-data two models were used. The first model simulated blinks by means of linear regression, and the second model simulated blinks by a component model. For both simulations the same data pool as in the real data analysis was used. One problem with the simulation of eyeblinks is the choice of an appropriate model for construction. Since it is not desirable to favour a particular correction by the type of calculations that are necessary for the simulation, blinks were both realized by a component model (i.e. assuming a linear mixture of statistically independent components) and a propagation model (i.e. adding blinks to clean data by linear regression). Both types of simulated data were analysed with respect to the correlation between clean, uncontaminated data and corrected data.

#### Propagation model

For the propagation model one subject was chosen randomly. From this subject the vertical EOG and eight randomly chosen channels were used for simulation. As first step, eyeblinks were detected by a threshold algorithm, and the vertical EOG was averaged time-locked to the maximum blink excursion to receive a blink template (temp). Subsequently eight random numbers in the range from −1:−1 were defined as propagation factors (p). The propagation factor for the vertical EOG channel (channel 9) was set to one, that yields a vertical EOG-channel showing typical blink artefacts. Next, the channels were manually cleaned from real eye blink activity. This was done by manually inspecting and removing all time points of blink activity. This dataset was now contaminated with eyeblinks by calculating back the eye blink by linear regression. This was done by multiplying the propagation factors (p) with the blink template (temp) and adding it to the cleaned EEG for each channel *c* and time point *t*:
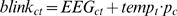



This procedure simulates eyeblinks with almost identical amplitude and duration. However, in real data spontaneous eyeblink amplitudes usually vary due to factors like eye dryness, fatigue, or cognitive load. Hence for a more realistic simulation a second regression simulation was conducted in which the simulated blinks had a randomly varying amplitude. This was realized by randomly enhancing the activity in the blink template in a range between 70 and 280 µV. These values were chosen since they resemble the variation in the real data (average blink amplitude real data: 215.28 µV; s = 40.29).

#### Component Model

The component-based simulation was conducted in several steps. Here also nine channels were simulated. At first a subset of nine subjects was chosen randomly without replacement from the 17 subjects. For simulation of cerebral sources, from each of eight subjects one independent component activation was chosen randomly. For simulation of a blink source the component resembling a typical blink artefact was chosen from the remaining subject. This leads to truly independent components. To simulate eyeblink contaminated signals these activations (cerebral, blink) were now mixed by a random square matrix (i.e. backprojection, the blink component was projected to one channel with a mixing coefficient of .95, this results in a channel representing a vertical EOG channel). To receive blink free reference data, the component representing the eyeblink artefact was set to zero according to the procedure described in the introduction. For each simulation 50 eyeblinks were simulated (average blink amplitude: 228.25 µV; s = 83,76). Both data sets consisted of 10^5^ data points, respectively. Finally both correction procedures were applied to the simulations and the clean, blink-free data was compared with the corrected data. Here the same measures were calculated as with the real data: The ERPs were time-locked to the maximum blink excursion, and the correlation between clean, uncontaminated data and corrected data was estimated (after normalization of the data).

## Supporting Information

Animation S1 Average devolution of activity during eye blinking subsequent application of EMCPs, EMCP w/s and extended infomax. Top: Topographic maps (spherical spline interpolation). Bottom: Blink-related vertical EOG. Note the large residual activity for EMCPs at time-point zero and the positivity occurring at about 250 ms following the maximum blink excursion. The vertical line indicates the current time-point.
(0.87 MB MPG)
